# Brief review on systematic hypothermia for the protection of central nervous system during aortic arch surgery: a double-sword tool?

**DOI:** 10.1186/1749-8090-6-153

**Published:** 2011-11-20

**Authors:** Haralabos Parissis, Umar Hamid, Alan Soo, Bassel Al-Alao

**Affiliations:** 1 Cardiothoracic Department, Royal Victoria Hospital, BT12 6BA, Belfast, UK & Northern Ireland

## Abstract

Antegrade selective cerebral perfusion in conjunction with hypothermia attenuate postoperative neurological injury, which in turn still remains the main cause of mortality and morbidity following aortic arch surgery. Hypothermic circulatory arrest however could be a useful tool during arch surgery, surgery for chronic thromboembolic disease, air on the arterial line during CPB, during cavotomy for extraction of renal cell carcinoma with level IV extension, or when dealing with difficult trauma to the SVC or IVC. Cerebral protective effects with hypothermic procedures including inhibition of neuron excitation, and discharge of excitable amino acids, and thereby, prevention of an increase in intercellular calcium ions, hyperoxidation of lipids in cell membranes, and free radical production.

The authors are briefly discussing the fundamental principles of using hypothermia as an adjunct tool of the cardiothoracic surgeon's practice. The relationship between temperature, flow, metabolic requirements and adverse effects is addressed.

## Background

The methods of brain protection during interruption of its blood supply have been refined and at least the last decade the mortality for complex arch surgery has dropped to less than 10% with an accompanied incidence of neurological damage of 5 to 6%. [1]. More impressively in a large series of total arch replacement with prolong circulatory arrest times, Sasaki et al have quoted a low mortality of 1.9%, and a low incidence of postoperative transient stroke of 6.6% and permanent stroke of 1.6% [2]

Few minutes of complete global ischaemia will cause neuronal death in normothermic brain. Systemic hypothermia with circulatory arrest is a clinical term used for nasopharyngeal low temperatures, possibly below 20 degrees. It is known that oxygen consumption decreases by 50% for every 10 degrees decrease in body temperature [3], thus hypothermia reduces the metabolic rate of the central nervous system and lengthens the period of induced or accidental brain ischemia.

They have been different school of thoughts regarding optimal body temperature, perfusate temperature and the way of delivery of the perfusate to the brain during hypothermic circulatory arrest, with a reasonable aim by some authorities [4] to achieve electroencephalogram silence as a threshold point of neuro-electrochemical relaxation and also to achieve oxygen saturation at the point of the jugular venous bulb above 90 to 95%.

Regarding retrograde delivery of cerebral perfusion during circulatory arrest, most of the efforts to document improvement in cerebral metabolic function as a result of retrograde flow have failed. These studies only observed advantages related to the removal of emboli from the arterial system. [5,6].

### Hypothermia: The tool

The nervous system has a high metabolic demands and limited energy stores, which make it vulnerable to ischemia. More specifically, the human brain accounts for 2% of the total body weight but is supplied by 15% of the cardiac output and consumes 20% of the total body energy expenditure at rest. The energy expenditure of the brain, can be divided into two components: the 'activation metabolism' requires 55% of total brain energy consumption and is directly related to cell activity; and the 'residual metabolism' consumes the remaining 45% of energy to ensure basal functions essential to cell survival in the absence of any activation. To meet its metabolic requirements, the brain converts glucose provided by glycogenolysis in the liver and in the muscles into ATP, the directly available source of energy. In the presence of oxygen, the oxidation of one molecule of glucose generates 38 ATP molecules through the glycolysis process, the citric acid cycle and the electron transport chain. ATP can also be generated from creatine kinase and adenylate kinase activities. In the absence of oxygen, the conversion of pyruvate into lactate yields to the production of two ATP molecules for each molecule of glucose metabolized. In addition, the brain can metabolize ketone bodies, glycerol, fatty acids and amino acids. The metabolic regulation of cerebral blood flow is depending on chemical and neurogenic factors. Chemical vasodilating factors include adenosine, nitric oxide, hydrogen and potassium ions. Neurogenic control consists of extrinsic and intrinsic regulations responsible for vasoactive effects and modulation of the cerebrovascular tone. During hypothermia there is an increase in glutamate and lactate concentrations. Glutamate is an excitatory neurotransmitter that has been shown to cause irreversible neuronal injury during ischemia if excessive amounts are released into the extracellular space, or if its reuptake is inhibited. Glutamate promotes the entry of calcium and sodium into neuronal cells [7]. It has also been shown that neuronal nitric oxide synthase mediates neuronal necrosis after hypothermic circulatory arrest and plays an important role in neurotoxicity. Inhibition of neuronal nitric oxide synthase substantially reduces neuronal cell death and results in clinically improved neurologic functions [8]. Although hypothermia protects the brain by decreasing the release of excitatory amino acids and lessening various detrimental enzymatic reactions, the cerebral system is extremely sensitive to ischemia. In addition, though, ischemia and hypoxia stimulate active responses in the brain, which persist long after substrate delivery has been restored. These responses include activation of transcription factors which up-regulate expression of genes contributing to apoptosis and inflammation, inhibition of protein synthesis, sustained oxidative stress, and neurogenesis.

Animal studies showed a drop of the brain metabolic rate of 50% at 28°C & 19% at 18°C [9].

The reduction of metabolic rate in relation to temperature exhibits an exponential curve [10], with a greater drop at high temperatures (about 6% for 1°C around 37°C) than at low temperatures (about 1% at 15°C).

Assuming a safe ischemic period of 4-5 min during normothermia, the safe period [11] of circulatory arrest for the central nervous system would not exceed 25 minutes at 18°C.

The rate of re-warming from hypothermic conditions should be slow, without excessive increase of blood flow temperature (maximum temperature difference of 5-10°C). With rapid re-warming sudden increase of cerebral blood flow due to decreased cerebrovascular resistance leads to increased cerebrovascular volume and cerebral edema. Moreover carbon dioxide (CO2) is considered to be one of the factors affecting cerebral blood flow during hypothermic extracorporeal circulation. Elevated levels of CO2 in blood may cause cerebrovascular dilation. For this reason, some centers use a method to improve cerebral oxygenation by providing CO2 during re-warming from hypothermic conditions. In addition, levels of PaCO2 in arterial blood are primarily responsible for regulation of pH. To regulate pH during hypothermic extracorporeal circulation, two methods are available: using alfa-stat or pH- stat. Using pH-stat, one restores pH by adding CO2 which might increase cerebral blood flow due to vasodilation, but it may increase cerebral edema and cause gas microembolization. [12]. Therefore, in clinical practice the alfa-stat method has been widely used which allows the pH to change with the incremental reduction of temperature as the buffering capacity for H increases; this resulting in mild alkalosis.

Another issue during cerebral protection is the determining of the ideal level of systemic hypothermia. Moderate (> 24°C) versus deep hypothermia, has been questioned. This is based on the assumption that Cerebral oxygen consumption decreases by 50-60% at a core temperature of 25-28°C, and further cooling does not decrease brain oxygen consumption [13]. This is opposed by findings reported on animal studies whereby the brain metabolic rate has dropped to 19% at 18°C [9]. Moreover, the regional cerebral blood flow with antegrade perfusion decreases to 62% of baseline at 28 and to 36% at 18°C [14]. Based on the previous findings, various studies [15-19] have shown comparable results when moderate or deep hypothermia is used however Kazui et al [20] indicates that for prolonged periods of circulatory arrest > 60 minutes, deep hypothermia (< 22°C) may be safer in terms of brain and spinal cord protection.

### Anatomical Variations of the Blood supply to the brain

The left and right internal carotid arteries arise from the right and left common carotid arteries. The posterior communicating artery is given off as a branch of the internal carotid artery just before it divides into its terminal branches - the anterior and middle cerebral arteries. The anterior cerebral artery forms the anterolateral portion of the Circle of Willis, while the middle cerebral artery does not contribute to the circle. The right and left posterior cerebral arteries arise from the basilar artery, which is formed by the left and right vertebral arteries. The vertebral arteries arise from the subclavian arteries. The anterior communicating artery connects the two anterior cerebral arteries and could be said to arise from either the left or right side.

In various studies only 34% of patients have been found to have a complete circle of Willis. Various anatomic variation exists as proposed by Lazorthes et al [21]. Therefore instead of one anterior and two posterior communicating arteries, one or two may be absent. As per Merkkolla et al [22] 22% of the anterior communicating arteries and 46% of the posterior communicating arteries were missing during Post mortem examinations. Theoretically, the absence of one of three communicating arteries does not carry any risk for hypoperfusion because the blood coursing through the right upper brachial artery will perfuse the whole brain through the vertebral, basilar, and internal carotid arteries. Again if hypothetically there is no cerebrovascular atherosclerotic disease present, the only combination that will carry the potential for contralateral hypoperfusion would be the absence of both anterior and posterior communicating arteries, and even if that is the case, only the frontal and temporal regions of the left hemisphere would be affected.

Nevertheless, these variations can have important clinical impact in selective cerebral perfusion during hypothermic arrest. This concept brings about the argument of unilateral versus bilateral antegrade cerebral perfusion. The use of bilateral transcranial Doppler for continuous measurement of blood velocity of the middle cerebral artery or the use of near-infrared spectroscopy for the estimation of the left hemispheric perfusion, are helpful tools during the decision making process [23].

### Hypothermia & Flow (unilateral versus bilateral)

Normally, basal cardiac output is determined by oxygen consumption, which is approximately 250 mL/min. The accepted flow rate at 37° and hematocrit of 25% is approximately 2.4 L/min m^2 ^in an anesthetized patient. As a general principle it is being recommended during hypothermia [24] that flows should be reduced only to levels, which permit at least 85% of maximal oxygen consumption. At 30°C this flow rate is approximately 1.8 L/min/m^2^; at 25°C, 1.6 L/min/m^2^; and at 18°C, 1.0 L/min/m^2^.

Deep hypothermia alone may be an adequate means of cerebral protection when the circulatory arrest time is less than 30 minutes [11].

The combination of hypothermia with low ante grade cerebral flow [25] provides additional brain protection. Deep hypothermia and low antegrade cerebral flow (0.5 lt/min per m^2^, or by maintaining the mean right radial pressure between 50-60 mmHg)) are used when blood flow to the brain must be interrupted for an arch reconstruction, or when a complex procedure is anticipated. By implementing such methods during circulatory arrest, the actual arrest time could be safely prolonged up to 90 minutes [26]. More specifically, there are some evidence that unilateral and bilateral antegrade brain perfusion resulted in neurological injury rates of less than 5% [27], however unilateral perfusion allows the safety arrest time to be around 30-50 minutes were the bilateral approach prolongs the safety time to above 80 minutes with an acceptably low rate of stroke [28]. Bilateral cerebral perfusion, while it provides greater time for complex and extensive aortic procedures, the main drawbacks are that it may clutter the surgical field, injure the arch vessel related with cannulation, and increase the risk of particulate embolization.

On the other hand the rare case of complete interruption of the right internal carotic artery due to atherosclerotic disease, renders right side antegrade cerebral perfusion detrimental and inadequate.

### Cerebral electrical activity

Theoretical complete suppression of cerebral electrical activity (EEG silence) should be an important goal in brain protection when a significant period of cerebral ischemia is anticipated. In the clinical setting, EEG ceases at a mean nasopharyngeal temperature of 17.5°C [29]. During this period the jugular venous bulb O2 saturation is measured > 90% due to the reduction of brain cellular extraction.

### Indication for the use of deep hypothermia

In order to answer the question as to how to optimally protect the brain while providing surgical access to the head and neck vessels is still unclear, due to lack of randomize control trials. Nevertheless it is apparent that the use of hypothermia combined with various forms of antegrade cerebral perfusion consists the base of cerebral protection.

Hypothermia is also indicated during accidental air introduction in the arterial line; the reason being that the solubility of air particles in a liquid is higher at low temperatures.

Some authorities [30] advocate profound hypothermia during thoracoabdominal surgery in order to enhance spinal protection.

### Deleterious effects of hypothermia

During hypothermia the blood viscosity is increased; this phenomenon is counteracted with haemodilution.

Low body temperature shifts the hemoglobin saturation curve to the left. This increases the affinity and impairs the release of O2 to the tissues.

Finally hypothermia interferes with enzyme and organ function, aggravates bleeding, increases systemic vascular resistance, delays cardiac recovery, lengthens duration of bypass, increases the risk of cerebral hyperthermia, and is associated with higher levels of depression and anxiety [31].

### Hypothermia and incidence of a stroke

By enlarge, the great majority of patients can be supported unharmed under a circulatory arrest time of 30 minutes at 18°C, provided that EEG silence is achieved. Various reports indicate that neurologic problems fall into 2 main categories. Temporary neurologic dysfunction, ranging clinically from simple disorientation, lethargy, or confusion at the mild end to prolonged extreme agitation or psychotic behavior at its most severe, has been reported in up to 25% of patients who undergo hypothermic circulatory arrest [32]. The occurrence of temporary neurological dysfunction is associated with the duration of the circulatory arrest and the method of brain protection [1]. Unfortunately, many reports have neglected temporary neurologic dysfunction events, by limiting neurologic evaluation to overt postoperative stroke, which has an incidence up to 10% [33]. Early stroke following surgery of the aorta may develop either by embolism or by hemodynamic mechanisms. Delay stroke (up to post operative day nine) can be due to hypercoagulation embolism from a calcific aorta or arch vessels.

Neurologic injury presents mostly as a focal deficit. A focal deficit is usually embolic due to micro-material or gas bubbles. Less frequently, a prolonged suboptimal perfusion of the brain can result in a localized necrosis in the transition area between two vascular territories (the so-called watershed lesion). The clinical picture includes motor-sensory deficit, aphasia, or cortical blindness.

The prevalence of a focal deficit in clinical series where deep hypothermia is used ranges from 5% to 10% [34]. Cerebral embolism can occur from the cannulation sites because of atherosclerotic changes in the ascending aorta near the arch aneurysm or during retrograde perfusion via the femoral artery. The theoretical advantages of using the right axillary artery site for inflow during complex aortic procedures have recently become apparent [35]. The authors concluded that with the use of an axillary access there is a decreased likelihood of stroke from embolic material, less likely malperfusion with aortic dissection, reduced disruption of atheroma or calcified plaques, and the ability to administer antegrade brain perfusion.

As per Ergin et al [36] incremental risk factors for the development of neurological injury include age, atherosclerotic aortic disease, manipulation of the aorta and the duration of hypothermia and circulatory arrest. Interestingly the type of repair (hemi-arch versus total arch repair) does not appear to influence neurological outcome. Furthermore, Immer et al [33] showed a reduction of transient neurological dysfunction with the use of antegrade cerebral perfusion during hypothermic arrest. Patients treated with selective antegrade cerebral perfusion had a higher quality of life during follow-up than did those with other methods of cerebral protection [37].

Finally, when compare the incidence of postoperative stroke between unilateral and bilateral brain protection in a propensity matched group of patients Olsson et al [38] showed a higher incidence of stroke after unilateral cerebral perfusion.

### Take home message

During hypothermia oxygen requirements in brain tissue are reduced. Cerebral protective effects during hypothermia, prevents the increase in intercellular calcium ions, hyperoxidation of lipids in cell membranes, and free radical production. Moreover, cell membranes are stabilized, and cellular and cerebral edema, caused by increased permeability in the blood-brain barrier, are inhibited. However, deep hypothermia alone may only be an acceptable means of brain protection when the anticipating arrest time is less than 30 minutes; furthermore antegrade cerebral perfusion by means of at least right hemisphere brain perfusion should be considered when the anticipated ischemic time is up to 50 minutes. Moreover when the transcranial Doppler identifies incompleteness of the circus of Willis or when the arrested ischemic brain time is anticipated to be prolonged (more than 80 minutes) then one should considered the need for bilateral cerebral perfusion [39].

Finally, deep hypothermia < 20°C is an important tool for any surgical work that involves interruption of the brains blood supply. Low temperatures during complex aortic surgery, is a surgeon's alias providing a thoughtful process, supports the indications of its use. Moreover, efficient patients monitoring and adherence to time limitations with arrest times no longer than 90 min, should preclude adverse outcomes.

## Competing interests

The authors declare that they have no competing interests.

## Authors' contributions

HP participated in the sequence alignment and multiple drafts of the manuscript, UH carried out the literature research, AS was helpful in literature review and the "discussion" part of the paper and BA assist in the development of the manuscript and advised on valuable amendments. The authors read and approved the final manuscript.
